# Two Distinct Clinical Courses of Human Cowpox, Germany, 2015

**DOI:** 10.3390/v9120375

**Published:** 2017-12-07

**Authors:** Ines Eder, Patrick Vollmar, Martin Pfeffer, Philipp Naether, Arne Christian Rodloff, Hermann Meyer

**Affiliations:** 1 Institute of Medical Microbiology and Epidemiology of Infectious Diseases, University Hospital, 04103 Leipzig, Germany; ines.eder@medizin.uni-leipzig.de (I.E.); acr@medizin.uni-leipzig.de (A.C.R.); 2 Central Diagnostic Laboratory Division, Bundeswehr Institute of Microbiology, 80937 Munich, Germany; patrickvollmar@bundeswehr.org; 3 Institute of Animal Hygiene and Veterinary Public Health, University of Leipzig, 04103 Leipzig, Germany; pfeffer@vetmed.uni-leipzig.de; 4 Ear, Nose and Throat Clinic, University Hospital, 04103 Leipzig, Germany; philippnaether@googlemail.com; 5 Department of Viruses and Intracellular Agents, Bundeswehr Institute of Microbiology, 80937 Munich, Germany

**Keywords:** cowpox virus, Orthopoxvirus, skin lesion, zoonotic transmission

## Abstract

Here we present two cases of human infection with cowpox virus with distinct clinical courses. A series of clinical photographs documents lesion progression over time. In the first case—an unvaccinated young veterinary assistant—a pustule was treated locally with cortisone. The lesion turned into a large ulcer accompanied by severe lymphadenitis. Based on her close contact to a sick stray cat, infection with cowpox virus was assumed and confirmed by virus isolation, PCR, and serology. The clinical course took up to eleven months until healing of the wound was complete. Transmission of cowpox virus from the cat was likely because a skin swab was PCR-positive and the cat had a high titer of anti-orthopoxvirus antibodies. In contrast, a rather mild clinical course of cowpox was confirmed in a 49-year-old male farmer vaccinated against smallpox. Only a small eschar developed, and wound closure was complete after 6 weeks.

## 1. Introduction

Since there is no more mandatory smallpox vaccination, an increasing proportion of the world’s population is now immunologically naïve against infections with orthopoxviruses. Additionally, with increasing numbers of immunocompromised individuals, the risk of such infections has increased even further [1,2]. A re-emergence of orthopoxvirus infections in different regions of the world, including feral vaccinia virus strains, endemic in India and South America, and monkeypox virus, which emerges in various parts of the African tropical rainforest, has been observed. In addition, infections with cowpox virus (CPXV), a member of the genus Orthopoxvirus (OPV), family *Poxviridae*, are observed in Europe in humans and in a broad range of animals, including cats, dogs, primates, elephants, and various zoo animals [3]. Wild rodents are believed to be the primary hosts and reservoirs of CPXV, and hunting cats are infected through their prey [4]. Humans usually acquire infection via contact with infected cats or pet rats [5]. In patients without underlying disease, CPXV infections manifest as mild skin disease and typically single pock-like lesions heal within 3–6 weeks. However, little is known about factors resulting in severe and prolonged clinical courses. Underlying immunosuppressive diseases contribute to a generalization, and in one patient a lethal outcome was reported [6].

## 2. Case Reports

### 2.1. Case 1

**Figure 1:**
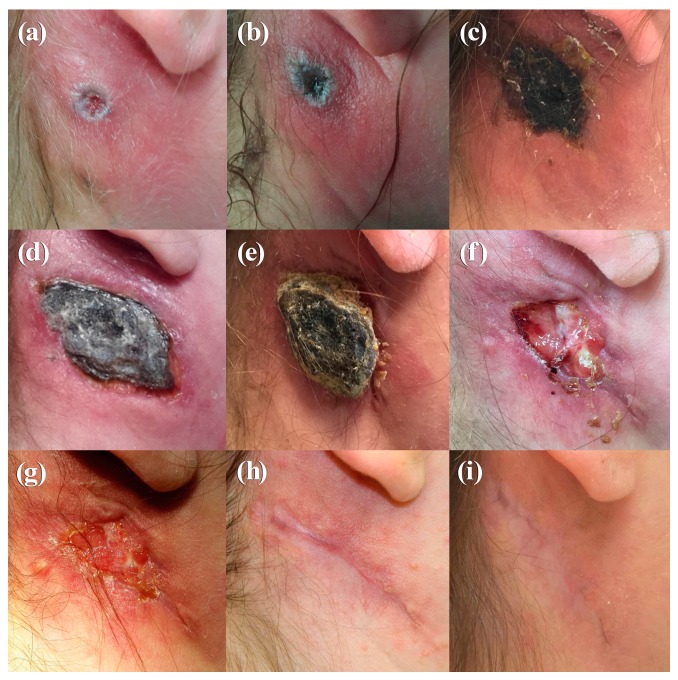
Photographs of the clinical course of cowpox in a veterinary assistant (Case 1), taken on Day 10 (**a**), Day 13 (**b**), Day 20 (**c**), Day 30 (**d**), Day 47 (**e**), Day 84 (**f**), Day 89 (**g**), Day 210 (**h**), and Day 326 (**i**).

A 23-year-old female veterinary assistant with no history of smallpox vaccination presented on 11 August 2015, at a dermatologist with a pustule behind her right ear and swollen cervical lymph nodes. A mosquito bite was suspected and oral Cefaclor and local cortisone ointment were applied. Ten days after onset, the ulcer had exacerbated and lymphadenitis was more severe (Figure 1a). Because an abscess was suspected, ampicillin-sulbactam treatment was initiated. The ulcerated skin lesion was accompanied by painful swelling and erythema (Figure 1b). Except for a pre-existing asthma that had been treated with cortisone spray; the patient was in good general condition without fever. Due to her contact with rabbits at work, an infection with the bacterium *Francisella tularensis* was suspected and antibiotic therapy was changed to ciprofloxacin. Ultrasound examination and computer tomography confirmed a lymphadenitis cervicalis, but no indication of an abscess was found. To rule out other differential diagnoses, skin swabs were investigated which resulted in growth of normal skin flora only. Serology turned out negative for lues, toxoplasmosis, HIV, Varicella zoster virus, Cytomegalovirus, Epstein-Barr virus, cat-scratch disease, rickettsiosis, and leishmaniosis. Because the ulceration developed a central necrosis (Figure 1b), infection with *Bacillus anthracis* was ruled out by PCR and serology. Tests to detect *Francisella tularensis* turned out negative as well. A more in-depth analysis of the patient’s medical history revealed close contact to a sick stray cat she had been caring for over the last weeks. The cat had ulcers on its paw and ear and swollen lymph nodes. Based on the clinical presentations of both, the veterinary assistant and the cat, a zoonotic infection with CPXV was assumed. Skin swabs obtained on Day 12 were transferred to the Central Diagnostic Laboratory Division of the Bundeswehr Institute of Microbiology. Inoculation on African green monkey kidney (MA104) cells [7] led to virus isolation. DNA extracted from the swab and the virus isolate were OPV-positive in the RealStar-Orthopox LC PCR Kit (Altona Diagnostics, Hamburg, Germany) [8]. Identity as cowpox virus was demonstrated by full-length sequencing of the two isolates (Ger/2015/Human2 and Ger/2015/Human2), as described recently [9] OPV-specific IgG antibodies with a titer of 80 were present on day 12 as shown by an immunofluorescence assay using vaccinia virus–infected MA104 cells. Skin swab and serum were taken from the cat. Although the ulcers were in the final stage of healing, the swab was OPV-PCR-positive; however, no virus could be isolated. In combination with a high titer of anti-OPV-antibodies (1280), these findings point to a recent CPXV infection in the cat. Antibiotic treatment of the patient was stopped and the ulcer was covered with a plaster to avoid autoinoculation and potential dissemination to patient contacts. On Day 20, the ulceration turned into an eschar (50 × 35 mm) (Figure 1c), and on Day 21 the patient was released from the hospital. The eschar extended to a size of 60 × 40 mm with deep necrosis (Figure 1d) and remodeled into a hyperkeratotic necrotic tissue (Figure 1e). Eighty-three (83) days after the onset of infection, the eschar finally fell off (Figure 1f). After secondary wound healing (Figure 1g), a 60-mm-long scar developed after an overall duration of 326 days (Figure 1h,i). The antibody titer increased from 80 on Day 12 after the detection of the lesion to a titer of 640 on Day 30 and declined to 160 after 210 days (Table 1).

### 2.2. Case 2

**Figure 2:**
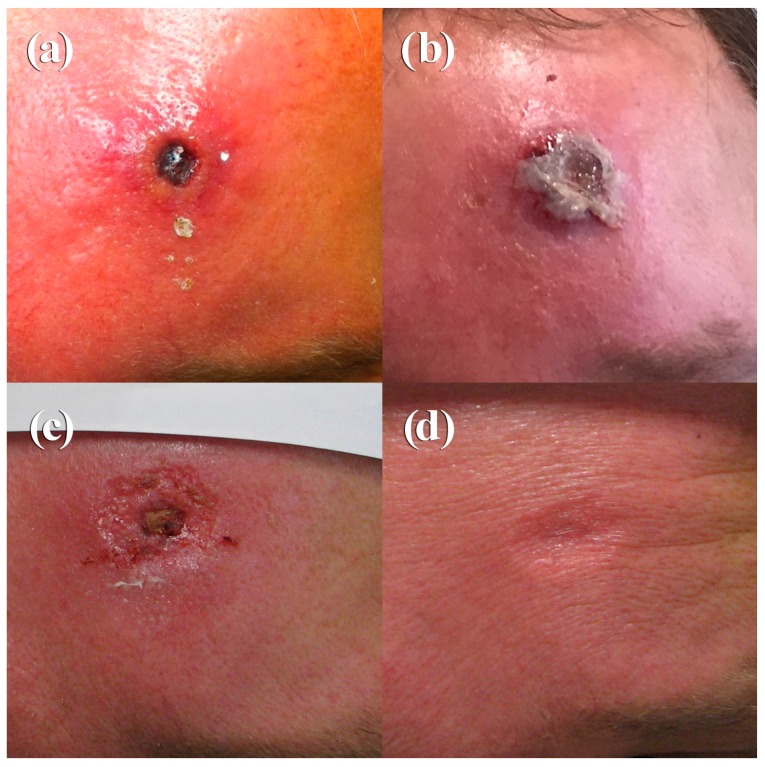
Photographs of the clinical course of cowpox in a 49-year-old farmer (Case 2), taken on Day 10 (**a**), Day 12 (**b**), Day 32 (**c**), and Day 41 (**d**).

A 49-year-old male farmer presented on 10 October 2015 with an ulcerated skin lesion on his forehead (Figure 2a). Ten days prior to admission, he had noticed the formation of a small fluid-filled pustule. Cervical lymph nodes were swollen and the phlegmonous inflammation on the forehead was very painful. He was treated with ampicillin/sulbactam and local oxytetracyclinhydrochlorid ointment. Routine microbiology diagnostics showed growth of normal skin flora, tularaemia, and anthrax diagnostics were negative. Since he was treated at the same department as Case 1 and thus taking benefit from a raised awareness, infection with CPXV was suspected based on the clinical presentation. A skin swap was taken and, by applying two PCR-assays followed by sequencing of the amplicon, CPXV-specific sequences were identified. Virus isolation was successful. On Day 10, a high titer of OPV-specific IgG antibodies (1280) was present. Analysis of the patient’s medical history revealed no contact with cats, but frequent contacts with dogs, rabbits, sheep, and pigs. The patient had been vaccinated against smallpox in 1978. Recently, he had performed pest control measures in his house and stables and he had direct contact with dead rodents, which could possibly have been the source of infection. Compared to Case 1, he developed a rather mild course with formation of a small eschar and secondary wound closure after 41 days (Figure 2b–d). At this time, the antibody titer had increased to 2560 (Table 2).

## 3. Discussion

A typical, localized course of cowpox is characterized by an inflamed macule that passes through papular and vesicular stages over a 7–12-day period. Systemic symptoms, such as elevated temperature, malaise, vomiting, and sore throat, are frequently reported [10]. After 2–3 weeks, a hard black eschar with edema, erythema, and induration evolves. Painful lymphadenopathy is sometimes described. Healing typically takes 4–6 weeks. This classical course was seen in the 49-year-old patient (Case 2) and is documented in Figure 2a–d. The fact that the patient had been vaccinated 37 years ago could have contributed to a milder disease course as also observed by Campe et al. [5] and to the rather high level of anti-OPV antibodies measured. In contrast, in the case of the unvaccinated veterinary assistant (Case 1), a much longer and more severe course was observed (Figure 1a–i). Whether (i) the location of the initial lesion in rather soft tissue, (ii) a possible higher amount of virus inoculated, or (iii) the local application of a cortisone-containing ointment had an influence on the outcome is a matter of speculation. Staff of the veterinary practice, who were all unvaccinated against smallpox and had handled this particular cat, did not show any lesions. This speaks for an individual predisposition or pre-existing skin lesion/injury through handling the cat and inoculating the virus. In Case 1, an apparent misdiagnosis was made initially, and the patient was placed on different courses of antibiotics. This is certainly of concern, especially in the context of the frequently reported emergence of antibiotic-resistant bacteria. Thus, in countries where OPV infections are re-emerging, in order to prevent wrongful use of antibiotics, it may be incumbent on healthcare policy makers to consider including early testing for OPV (e.g., CPXV in Europe) where a patient presents with symptoms and/or lesions that may be caused by CPXV. Based on a remarkably high number of diagnostic assays that had been conducted to rule out various differential diagnoses, it seems that physicians are not very familiar with CPXV infections. Therefore, in cases of poorly healing skin lesions, especially in immunocompromised patients [11,12,13], an in-depth medical and social history analysis is needed to rule out CPXV infection. As a result, ineffective therapies or even unnecessary surgical procedures can be avoided. In addition, there is a need for selective vaccination of workers who may be predisposed to exposure to OPV infections to minimize the risk of zoonotic infection. The two distinct clinical courses of human cowpox could not be linked epidemiologically although they occurred within 8–9 weeks (August and October 2015) and only 25 km apart. Comparison of the hemagglutinin gene sequences (942 bp) revealed a single mismatch only. However, since identical hemagglutinin sequences from Erfurt (1988), Munich (2001), and the larger Leipzig area (2000, 2012) are available in GenBank, sequencing of as little as 0.5% of the entire CPXV genome is not sufficient for setting up a molecular epidemiology. Sequences of the entire genome are needed. Currently, sequences of various CPXV strains are being determined to better understand the epidemiology of this zoonotic disease.

## Figures and Tables

**Table 1:** Human cowpox in a 23-year-old veterinary assistant over a period of 11 months.

Day	Figure	Clinic, Diagnostic Findings
Day 0		Small pustule retroauricular on the right side
Day 6		Pustule, lymphadenitis, local application of cortisone
Day 10	Figure 1a	Round ulceration with sharp margin (ca. 10 × 10 mm) and surrounding erythema and edema, painful swelling, lymphadenitis, abscess suspected, hospitalization
Day 11		Skin swab: Growth of normal bacterial skin flora
Day 12		Skin swab: Positive for Orthopoxvirus (OPV) DNA, anti-OPV-titer 80, virus isolation positive
Day 13	Figure 1b	Round to ovoid ulceration with central necrosis and surrounding erythema and edema
Day 20	Figure 1c	Ulceration turns into an eschar with still inflamed surrounding skin (ca. 50 × 35 mm)
Day 21		Discharge from hospital
Day 30	Figure 1d	Eschar at its maximum extend measuring ca. 60 × 40 mm with deep necrosis and prominent swelling of the margin of the wound
Day 37		Anti-OPV-titer 640
Day 47	Figure 1e	Eschar remodels into hyperkeratotic necrotic tissue and starts to flake off, surrounding erythema is progressive, anti-OPV-titer 320
Day 77		Anti-OPV-titer 320
Day 83		Eschar falls off, biopsy of eschar: OPV-PCR-positive, virus isolation negative
Day 84	Figure 1f	After flaking of the eschar a ca. 50 × 35 mm necrosis remains with incipient granulation of the wound and fibrin coating
Day 89	Figure 1g	Secondary wound healing with advanced granulation and fibrin coating, skin swab OPV-PCR-positive
Day 106		Anti-OPV-titer 320
Day 110		Ending of sick leave
Day 210	Figure 1h	Scar formation, remaining hyperpigmentation of the formerly inflamed surrounding tissue, anti-OPV-titer 160
Day 326	Figure 1i	A 60 mm long cicatrix remains

**Table 2:** Human cowpox in a 49-year-old farmer over a period of 6 weeks.

Day	Figure	Clinic, Diagnostic Findings
Day 0		Pustule on the forehead
Day 6		Painful swelling, lymphadenitis
Day 10	Figure 2a	Circular ulcerated wound with sharp margins and deep-seated eschar (ca. 10 × 10 mm) surrounded by erythema and edema Skin swab: Growth of normal bacterial skin flora Skin swab: Positive for OPV DNA, virus isolation positive Anti-OPV-titer 1280
Day 12	Figure 2b	ca. 12 × 12 mm eschar on still inflamed surrounding skin
Day 32	Figure 2c	Regressive redness and swelling of the skin, flaking of the eschar, still incrusted wound base with granulation in the surrounding tissue, Anti-OPV-titer 2560
Day 41	Figure 2d	Wound covered with epithelial tissue after secondary wound healing
